# Visual outcome of pars plana vitrectomy with intraocular foreign body removal through sclerocorneal tunnel and sulcus-fixated intraocular lens implantation as a single procedure, in cases of metallic intraocular foreign body with traumatic cataract

**DOI:** 10.4103/0301-4738.60077

**Published:** 2010

**Authors:** Santosh K Mahapatra, Nageswar G Rao

**Affiliations:** Vitreo Retinal Service, JPM Rotary Eye Hospital and Research Institute, Sec-6, CDA, Bidanasi, Cuttack, Orissa - 753 014, India

**Keywords:** Combined procedure, intraocular foreign body, pars plana vitrectomy, small-incision cataract surgery, traumatic cataract

## Abstract

**Aim:**

To evaluate visual outcome following pars plana vitrectomy (PPV) and intraocular foreign body (IOFB) removal through the sclerocorneal tunnel combined with simultaneous cataract extraction and sulcus-fixated intraocular lens (IOL) implantation as a single procedure in penetrating ocular trauma with IOFB and traumatic cataract.

**Materials and Methods:**

Eighteen cases of penetrating ocular trauma with retained IOFB and traumatic cataract who underwent PPV, IOFB body removal and cataract extraction with posterior chamber IOL (PCIOL) implantation in the same sitting, between June '04 and December '05 were retrospectively analyzed. All the foreign bodies were removed through the sclerocorneal tunnel.

**Result:**

All the 18 patients were young males, with an average follow-up period of 12 months. In 12 cases the foreign body was intravitreal and in six cases it was intraretinal but extramacular. Thirteen cases had a best corrected visual acuity ranging from 20/20 to 20/60 at their last follow-up. Five cases developed retinal detachment due to proliferative vitreoretinopathy (PVR) changes postoperatively and were subsequently managed by surgery.

**Conclusion:**

Primary IOL implantation with combined cataract and vitreo-retinal surgery is a safe option reducing the need for two separate surgeries in selected patients with retained IOFB and traumatic cataract. This combined procedure provides good visual outcome with early rehabilitation in young working patients.

Open globe injury with or without intraocular foreign body (IOFB) often has a poor visual outcome owing to possible endophthalmitis and/or vitreo retinopathy with the varied and unsterile wounds associated with it.[1–3] Management of IOFB in the presence of cataract and vitreoretinal pathology is obviously a challenge.[4–8] Removal of cataract is necessary for visualization of posterior segment, management of IOFB and early visual rehabilitation of the patient.

Methods for removal of the traumatic cataract include lensectomy, conventional extra-capsular cataract extraction (ECCE), manual small-incision cataract surgery (SICS) and phacoemulsification. Unlike lensectomy other methods of cataract extraction offer a better visual rehabilitation because of implanted intraocular lens (IOL).[5–8] Most surgeons remove the IOFB through the enlarged sclerotomy, but the enlargement of the sclerotomy carries a significant risk of hypotony, vitreous hemorrhage, peripheral vitreous incarceration into the wound intraoperatively and retinal detachment (RD) postoperatively.[2–4]

To avoid such complications, in this series we performed cataract extraction by manual SICS (6 mm sclerocorneal tunnel), removed the foreign body through the same tunnel and implanted a posterior chamber IOL (PCIOL) over the anterior capsular rim. The aim of the study was to evaluate visual outcome following pars plana vitrectomy (PPV) and IOFB removal through the sclerocorneal tunnel combined with simultaneous cataract extraction and sulcus-fixated IOL implantation as a single procedure in penetrating trauma with IOFB and traumatic cataract.

## Materials and Methods

Medical records of patients with penetrating eye injury and IOFB attending the retina clinic between June 2004 and December 2005 were reviewed. Out of 28 consecutive patients, 10 patients were excluded from this study because of absence of cataract, presence of associated endophthalmitis, severely injured lens with vitreous in the anterior chamber and evidence of zonular dehiscence.

**Figure 1:**
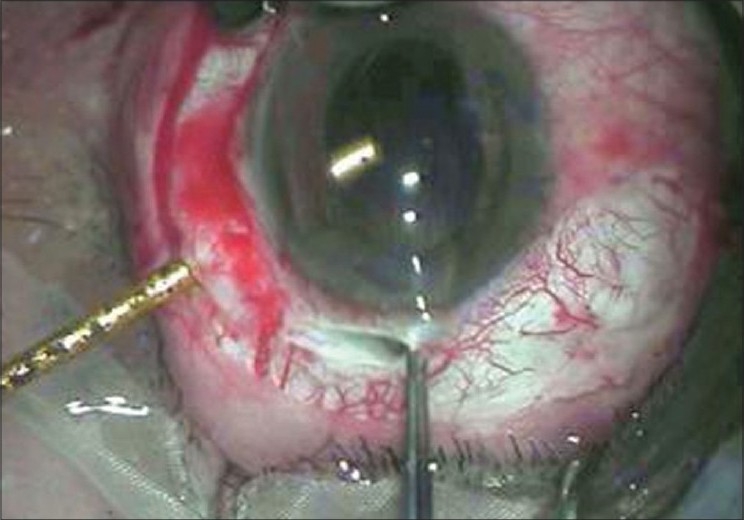
Removal of intraocular foreign body through scleral tunnel with utrata forceps from tip of earth magnet

**Figure 2:**
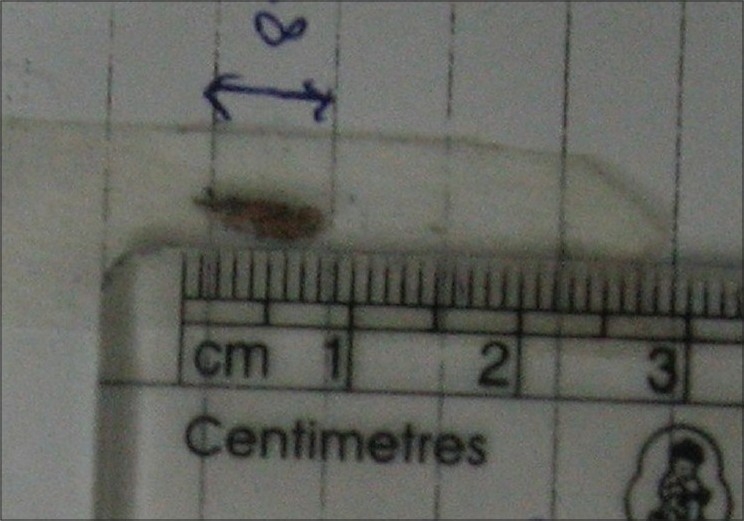
Photograph of a removed iron foreign body with measuring scale

**Figure 3:**
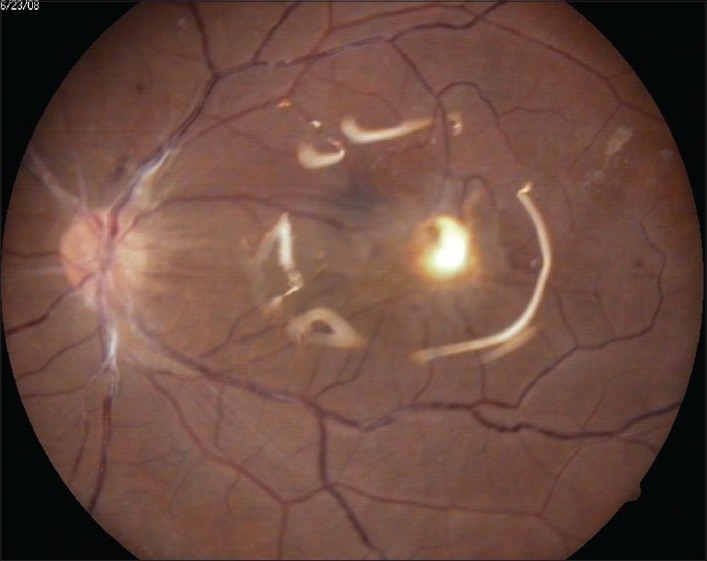
Attached retina with a para macular scar in an oil-filled eye after removal of intraocular foreign body

**Figure 4:**
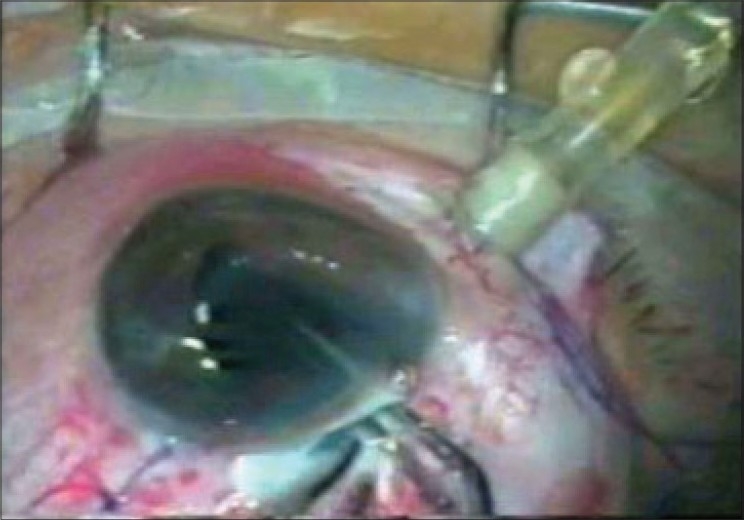
Intraocular lens being placed after removal of foreign body, over the anterior capsular rim

Eighteen patients with clinically significant lens opacification and IOFB with or without concomitant vitreoretinal pathology were included in the study. Primary repair was done in all cases except three, where the wound was self-sealed and they were taken up for vitreoretinal procedure directly. All patients underwent a complete general ophthalmologic examination prior to the surgical procedure. Preoperative visual acuity, site of entry of foreign body (FB), capsular and zonular integrity was assessed. Ultrasound B-Scan and/or X-ray of orbit were performed in all patients to locate and evaluate the IOFB. Keratometry and biometry of the injured eyes were done. If keratometry and biometry was not possible on the injured eye, fellow eye measurements were used. In all cases manual SICS was performed before the vitreoretinal procedure. A 6 mm frown-shaped sclerocorneal tunnel was made, followed by continuous curvilinear capsulorrexis and hydrodelineation. The lens nucleus was prolapsed into the anterior chamber and removed through the tunnel by sandwich method.[9–11] Rest of the lens material was removed by dry aspiration under viscoelastics. Scleral incision was temporarily closed with a single 10.0 nylon suture in McLean technique.[12,13] Standard three-port PPV was performed with a 20-gauge vitreous cutter and handheld light source. Posterior vitreous detachment was induced when it was not there. The FB was made free from its attachments. As all FBs were magnetic, they were lifted to the pupilary plane using an intraocular magnet and then Utrata forceps was used to grasp the FB and gently remove it through the sclerocorneal tunnel [Figs. 1 and 2]. Endolaser and retinal endotamponade [Fig. 3] was used in cases with a retinal break. PCIOL was implanted over the anterior capsular rim (in the sulcus) [Fig. 4]. The IOL was a single-piece polymethyl metha acrylate (PMMA) lens with 6 mm optic.

Sclerotomies and conjunctiva were closed with 6-0 vicryl and 2 mg dexamethasone and 2 mg gentamicin sulphate were injected subconjunctivally. Postoperatively all patients received topical antibiotics and steroid for four weeks with gradual tapering. Oral ciprofloxacin 500 mg twice daily along with nonsteroidal anti-inflammatory agents was given in all cases with addition of systemic steroids when necessary. In each case recording of best corrected visual acuity (BCVA), intraocular pressure measurement, slit-lamp biomicroscopy and indirect ophthalmoscopy was done postoperatively. Cause of decreased vision was assessed considering site of entry of FB, site of lodgment of FB and postoperative complications like RD in each case.

## Results

All our patients were young males with mean age of 24.5 years. The minimum follow-up period was four months, the maximum being 18 months with median follow-up time of 12 months. In 12 out of 18 cases the FB was intravitreal and in six cases it was intraretinal but extramacular. Thirteen out of 18 cases had BCVA between 20/20 and 20/60. Anatomical success was achieved in 13 out of 18 cases with 11 patients having visual acuity 20/30 or better with first vitreoretinal procedure. With the second vitreoretinal procedure four out of five patients had anatomical success [Table 1].

**Table 1:** Demographic and clinical profile of the patients

Age	Sex	Time to SX(days)	Entry site	Size of FB (mm)	Location of the FB	Average F/U period (months)	Pre-Op. VA	Final VA	Complications
16	M	10	Central corneal	3	Intravitreal	18	CF close	20/60	Nil
25	M	9	Para central corneal	4	Intravitreal	12	HM	20/60	Nil
32	M	45	Para central corneal	4	Intravitreal	18	HM	20/40	Nil
18	M	9	Corneal	3	Intravitreal	12	20/60	20/20	Nil
28	M	7	Para central corneal	2	Intravitreal	6	20/80	20/30	Nil
19	M	14	Scleral	6	Parsplana	24	CF 1 m	CF ½ m	RD
18	M	18	Corneal	3	Intravitreal	12	CF 1/2 m	20/60	Nil
40	M	10	Para central corneal	3	Retinal	16	HM	20/30	Macular RPE changes
24	M	15	Para central corneal	2.5	Intravitreal	18	HM	20/60	Nil
26	M	10	Corneal	2	Intravitreal	12	20/40	20/30	Nil
23	M	3	Para central corneal	3	Retinal	18	CF	CF 1 m	GRT with RD
35	M	14	Corneal	3	Intravitreal	8	HM	20/20	Nil
30	M	30	Corneo scleral	5	Retinal	12	PL+	CF ½ m	RD
30	M	4	Corneal	4	Paramacular	18	HM	20/40	Paramacular scar
20	M	30	Corneal	5	Intravitreal	12	HM	20/20	Macula on RD
12	M	12	Para central corneal	8	Retinal	6	PL+	HM	RD
20	M	30	Corneal	1	Intravitreal	12	20/200	20/30	Nil
26	M	60	Para central corneal	7	Intravitreal	18	HM	CF ½ m	RD

M - Male, mm - Millimeter, FB - Foreign body, F/U - Follow-up, VA - Visual acuity, CF - Counting finger, HM - Hand movement, RD - Retinal detachment, GRT - Giant retinal tear, SX - Surgery

## Discussion

We reviewed the visual outcome and complications in 18 patients with penetrating intraocular injury, significant cataract and retained IOFB, who underwent manual SICS, PPV, removal of IOFB and implantation of PCIOL. This simultaneous procedure was chosen to get rid of cataract, which diminished the visualization of posterior segment and for prevention of lens-induced uveitis due to a ruptured lens capsule. We found no significant correlation in ‘duration between injury and vitreoretinal procedure’ which is well supported by other studies.[3,14–19] But a gap of more than two months between injury and vitreoretinal procedure is mentioned as a poor prognostic factor by Yozo *et al*.[14] We had put a scleral buckle when the FB was intraretinal and lodged beyond mid-periphery or when complete base excision was not achieved. Role of prophylactic scleral buckle though supported by a few studies, is not established.[14–19]

Sclerocorneal tunnel and sclerotomy have their advantages and disadvantages as a route for removal of IOFB. Enlargement of sclerotomy carries risk of hypotony, vitreous hemorrhage, peripheral vitreous incarceration into the wound intraoperatively, and RD postoperatively. Removal of the FB through sclerocorneal tunnel may damage or sacrifice the integrity of the posterior lens capsule and anterior capsular rim, but large FBs are easily removed through the sclerocorneal tunnel.[14–19]

Except few isolated case reports with large IOFBs, so far there is no data on removal of cataract and FB through the same route. In our experience, IOFB can be safely removed through the sclerocorneal tunnel and IOL can easily be implanted into the cilliary sulcus. In our study, the final BCVA was 20/30 or better in seven out of 18 cases, between 20/40 and 20/60 in six cases and counting finger (CF) at 1 meter (1 m) or less in five cases. This was very well comparable with similar studies.[5–8,13,14] Azad *et al*.[5] reported final visual acuity of 20/30 or better in all three of their patients with IOFBs and traumatic cataracts, who underwent single-stage pars plana lensectomy with anterior capsule preservation, vitrectomy, removal of the FB, and IOL implantation. Tyagi *et al*.[6] reported visual acuity of 20/30 or better in eight out of 10 patients who underwent simultaneous cataract extraction, vitreoretinal surgery, removal of IOFB and posterior chamber IOL implantation for ocular trauma due to IOFB. Lam *et al*.[7] reported a BCVA ranging from 20/20 to 20/40 in all four patients in their study with combined phacoemulsification, PPV, removal of IOFB and primary IOL implantation. Batman *et al*.[8] reported BCVA improvement in 13 out of 17 eyes who underwent simultaneous clear corneal phacoemulsification, PPV, IOFB extraction and IOL implantation. In our study, 13 cases had more than two-line and 11 had more than four-line improvement in visual acuity. The reasons for moderately poor vision in our patients were i) central or para central corneal scar (8) ii) FB lodgment in the para macular region (2).

Late onset of RD in our series was seen in five out of 18 cases. The incidence of RD in the intraretinal FB group in this series was seen in five out of six cases. The possible causes of this overwhelming incidence of RD in the intraretinal FB group might be increased manipulation of FB and retinal penetration increasing the chance of proliferative vitreoretinopathy (PVR) changes postoperatively.[16] These changes either caused a traction tear postoperatively or reopening of treated retinal break and a late onset rhegmatogenous RD (RRD). Despite recent surgical advances, RRD remains a devastating complication after ocular injury with IOFB.[20–23] Several clinical series reported incidence of late RRD following successful IOFB removal ranging from 15-32%.[1–3,20–23]

Combined cataract and vitreous surgery with FB removal and IOL implantation offers not only faster visual rehabilitation[6] but also reduces the number of hospital stays.[24,25]

According to our clinical experience, the surgery that combines cataract extraction, IOFB removal and IOL implantation is a safe and desirable operation in patients with IOFB and significant lens opacities. The main advantage of this procedure is rapid visual rehabilitation with a single surgery, reducing the cost and patient discomfort. Randomized controlled studies involving larger number of patients with longer follow-up period may be needed to establish these facts.
